# Associations between neighborhood socioeconomic status with depressive symptoms, and psychological distress among Asian American adults in New York City

**DOI:** 10.1038/s41598-025-31535-8

**Published:** 2025-12-09

**Authors:** Suditi Shyamsunder, Dyuthy Ramachandran, Chaeyoung Kim, Jaiveer Singh, Shozen Dan, Malathi Srinivasan, Latha Palaniappan, Eugene Yang, Tali Elfassy

**Affiliations:** 1 Stanford Center for Asian Health Research and Education (CARE), Stanford University School of Medicine, Stanford, CA USA; 2 Perelman School of Medicine, University of Pennsylvania, Philadelphia, PA USA; 3 Division of Primary Care and Population Health, Stanford University School of Medicine, Stanford, CA USA; 4 Division of Cardiology, University of Washington School of Medicine, Seattle, WA USA; 5 Department of Medicine, Katz Family Division of Nephrology and Hypertension, University of Miami Miller School of Medicine, 1120 NW 14th street, Suite 822, Miami, FL 33136 USA

**Keywords:** Neighborhood, Socioeconomic status, Mental health, Asian american, Psychology, Public health

## Abstract

The relationship between neighborhood socioeconomic status (NSES) with mental health among Asian Americans (AA) is underexamined. We sought to determine whether NSES is associated with symptoms of psychological distress or depression among AA residents of New York City (NYC). We examined 4,557 Chinese, Asian from the Indian Subcontinent (ISC), or Other Asian participants of the NYC Community Health Survey, 2018–2020. Participants self-reported psychological distress using the (Kessler-6 (K6)) and depressive symptoms using the Patient Health Questionnaire-8 (PHQ8), with higher scores indicating worse mental health. The neighborhood was defined by residence in one of 55 districts. We constructed a NSES factor score from neighborhood levels of: unemployment, poverty, high rent burden, and a college or greater education. NSES was categorized into tertiles. Hierarchical linear models assessed associations between NSES and mental health, adjusted for individual level age, sex, income, education, nativity, body mass index, current drinking, current smoking, and physical activity. Among NYC AA residents, 52% were women, 45% were 25–44 years old, 25% had less than high school education, and 56% lived in poverty. Living in a high compared with low NSES tertile associated with a lower PHQ8 scores (beta: -0.65; 95% CI: -1.10,-0.19) among Chinese NYC residents and a higher K6 score (beta 1.27; 95% CI: 0.59,1.95) among Asians from the ISC NYC residents. In NYC, living in low NSES neighborhoods was associated with less depression symptoms among Chinese Americans, and greater psychological distress among Asians from the ISC. These results underscore that neighborhood context is associated with mental health and that aggregation of AA into one group can obscure important associations.

## Introduction

The US is experiencing a growing mental health problem. Exacerbated by the COVID-19 Pandemic, the prevalence of both anxiety and depression has increased^1^. In 2022, about one in five US adults reported symptoms of anxiety or depression, with a disproportionate impact on racial and ethnic minorities. For example, non-Hispanic Black adults are less likely to be treated for Major Depressive Disorders and often experience a more severe burden compared to Non-Hispanic White adults^2^. Similarly, Hispanic adults are less likely to access mental health resources when needed compared with other groups such as non-Hispanic White adults^3^. However, among Asian Americans, the fastest growing segment of the US population^4^, less is known about mental health relative to Non-Hispanic White, non-Hispanic Black, and Hispanic adults. Historically, Asian American health has been under-studied, in part due to the model minority myth) which posits that this demographic is socioeconomically advantaged and therefore enjoys a higher quality of life^5^. However, this narrative runs contrary to the reality for many Asian American groups.

In New York City (NYC), Asian Americans account for 15% of the population, and are disproportionately of lower socio-economic status (SES)^6^ with poverty rates that resemble or exceed that of other ethnic minority populations such as Black and Hispanic adults^7^. For example, in NYC, 24% of Asian American adults live below the federal poverty line compared to 13% of non-Hispanic White adults, 23% of non-Hispanic Black adults, and 26% of Hispanic adults^7^. It is well known that lower SES is associated with adverse mental health conditions^8^. In particular, there is evidence showing an inverse gradient between SES with depression and anxiety^9,10^. Given the link between SES and mental health, Asian Americans residing in NYC may be at greater risk of adverse mental health conditions.

Neighborhood Socioeconomic Status (NSES) refers to the collective socioeconomic status of a neighborhood often conceptualized by factors that include educational attainment, walkability, crime rates, and poverty level across a geographic region^11^. It is increasingly recognized that the built environment, specifically the neighborhood context, exerts an influence on health, including mental well-being above and beyond individual level factors^11^. For example, residents of resource-deprived neighborhoods have been shown to have poor health outcomes compared to residents of affluent areas^11^. On the other hand, moving to a better neighborhood is associated with improvements in mental health^12^. Yet, the relation between NSES and mental health outcomes has seldom been described among Asian Americans. Further, most research related to Asian American health has often failed to disaggregate Asian Americans by background, despite the substantial heterogeneity within the US Asian American population^13,14^. This study aims to fill these gaps and specifically, to determine whether NSES is associated with depression and psychological distress, with partial disaggregation of Asian American groups residing in NYC.

## Methods

### Study population

The NYC Community Health Survey (CHS) is a cross-sectional telephone survey conducted by the NYC Health Department that includes 8,000 to 10,000 adult New Yorkers each year that is publicly accessible (https://www.nyc.gov/site/doh/data/data-sets/community-health-survey.page). It has been conducted annually since 2000 and is representative of the NYC adult population as a whole. To obtain a representative sample of non-institutionalized adult New Yorkers, the CHS uses a dual frame sample design consisting of random-digit-dial landline and cellular telephone exchanges that cover NYC and incorporates a disproportionate stratified random sample design. The CHS is conducted in five languages including Mandarin and Cantonese. The study population includes 4,557 self-identified Asian participants of four CHS cycles (2017–2020) who were geolinked to their respective neighborhoods (defined below). All study participants provided informed consent and IRB approval was obtained at the NYC Health Department and the University of Miami. In addition, all methods were performed in accordance with the relevant guidelines and regulations.

### Neighborhood socioeconomic status

Neighborhoods were defined according to community districts which were retrieved from participants’ addresses. Briefly, community districts are mutually exclusive administrative units comprising roughly 3 zip codes. While there are 59 community districts in NYC, the NYC Health Department combined two sets of community districts in Manhattan and two sets in the Bronx for a total of 55 analytic community districts utilized in this study. Combined community districts had similar socio-economic characteristics. Socioeconomic information for each community district is publicly available through the NYC government (https://communityprofiles.planning.nyc.gov/). Specifically for each community district (defining the neighborhood), we extracted 4 measures based on the proportion of the community district: (1) unemployed, (2) living below the federal poverty line, (3) with a college or greater education, and (4) with a high rent burden (defined as households that spend 35% or more of their income on rent). Then, we used these four metrics to estimate an NSES factor score for each community district using the principal factor method with a loading threshold of 0.3^15^. The NSES factor score was subsequently categorized into tertiles representing low NSES (disadvantageous), middle NSES, or high NSES (advantageous).

### Mental health

*Depressive symptoms* and *non-specific psychological distress* were each assessed through questionnaires. *Depressive symptoms* were measured using the validated 8 item Patient Health Questionnaire (*PHQ-8*) depression scale in the 2017-18 CHS cycles. Briefly, the PHQ-8 is a series of 8 questions assessing frequency over the last two weeks that one reports being bothered by feelings of hopelessness, lack of energy, poor appetite, and other scenarios^16^. The score ranges from 0 to 24 with higher scores indicating higher levels of depressive symptoms^17^. The PHQ-8 has been shown to be non-inferior to the lengthier PHQ-9 form^18^, a lengthier version also used to screen for depression. *Non-specific psychological distress (NSPD)* was measured using the Kessler Screening Scale for Psychological Distress (*K6*) in the 2019-20 CHS cycles. The K6 is a 6-item screening scale assessing frequency of moods such as hopelessness and depression over the past 30 days^19^. The scores can range from 0 to 24 with higher scores indicating higher levels of psychological distress, and the K6 has been shown to be reliable and valid^20^.

### Other variables

Through survey questionnaires, participants self-reported their age in age groups (18–24, 25–44, 45–64, or 65 + years), sex, and Asian background (Chinese, from the Indian Subcontinent (ISC), or Other Asian). Asians from the ISC included all participants who descended from the ISC, such as India, Pakistan, Bangladesh, and Sri Lanka, encompassing a broad range of different nations, cultures, religions, and customs. Participants reported family size as the number of individuals per household, and reported their household income from all sources which was then used to estimate federal poverty level (FPL): less than 200%, 200–399%, or ≥ 400%. Participants also reported their educational attainment defined as less than high school (HS), HS graduate, some college, or college graduate or higher and their nativity status (US born or foreign born). Their self-reported heights and weights were used to calculate body mass index (BMI). BMI in kg/m^2^ was categorized according to Asian cutoff points as follows: under/normal weight (BMI: < 23 kg/m^2^), overweight (BMI: 23–27.4 kg/m^2^) or obese (BMI ≥ 27.5 kg/m^2^)^21^. Participants also self-reported whether they had consumed any alcohol in the last 30 days (current drinker), and whether they were current smokers (smoked at least 100 cigarettes in their lifetime and currently smoke cigarettes). To measure physical activity, participants were asked the following yes/no question, “during the past 30 days, other than your regular job, did you participate in any physical activity or exercises?”

### Statistical analysis

The data structure of this analysis includes 2 levels: 4,557 participants nested within 55 neighborhoods in level 2. First, we described the proportions of individual-level characteristics of the sample overall and according to Asian sub-group. We used chi-square tests to determine whether characteristics differed according to Asian sub-group. Next, we described neighborhood-level characteristics overall and across tertiles of NSES. Then, we estimated mean K6 and PHQ8 for each Asian sub-group across tertiles of neighborhood SES score. For each Asian sub-group (and each outcome), we used t-tests to determine whether means differed significantly from high NSES as the reference.

To determine whether NSES was associated with depressive symptoms and NSPD we fit hierarchical linear regression models. For each outcome we ran two models. Model 1 was adjusted for: age, sex, individual level poverty and education. Model 2 included model 1 covariates in addition to: nativity, BMI, current drinker, current smoker, and physical activity. We tested for a statistical interaction between NSES with Asian sub-group for each outcome of interest, and stratified models by Asian sub-group. We did not adjust for multiple comparisons due to an a-priori decision to disaggregate Asian sub-groups. All analyses incorporated survey weights, stratification design variables, and are representative of NYC as a whole. Analyses were conducted using SUDAAN (version 10.0; Research Triangle Institute, Research Triangle Park, North Carolina) and MPLUS (Version 7; Muthen and Muthen 1998–2012).

## Results

Most of the study population was 25–44 years old (45.1%), with 52.1% female, 55.9% living below 200% of the FPL, 25.0% having less than a HS education, and 14.2% born in the US, Table 1. Chinese residents of NYC tended to be older, have a higher poverty rate, have lower educational attainment, and less likely to be US born with Asians from the Indian sub-continent or Other Asians (p values < 0.05). Chinese residents of NYC were also least likely to reside in neighborhoods of high NSES compared with other Asian sub-groups (p-value < 0.05).

**Table 1:** Characteristics of Asian American residents of new York City, new York City community health Study, 2018–2020.

		Asian American Sub-Group			
	Overall (unweighted *N* = 4557)	Chinese (unweighted *n* = 2598)	Indian Subcontinent (unweighted *n* = 1,210)	All other (unweighted *n* = 749)	Chi-square
	% (CI)	% (CI)	% (CI)	% (CI)	*P*-Value
Characteristic					
Age group					
18–24	14.5 (12.9, 16.2)	12.4 (10.5, 14.6)	18.2 (14.9, 22.0)	17.3 (13.4, 22.0)	< 0.01
25–44	45.1 (42.9, 47.3)	41.6 (38.8, 44.5)	46.9 (42.6, 51.2)	56.6 (51.2, 61.9)	
45–64	27.9 (26.0, 29.9)	30.0 (27.5, 32.6)	27.8 (24.1, 31.8)	19.6 (15.8, 24.2)	
65+	12.5 (11.2, 13.9)	16.0 (14.1, 18.1)	7.1 (5.5, 9.2)	6.5 (4.5, 9.3)	
Female	52.1 (49.9, 54.3)	53.1 (50.2, 55.9)	48.4 (44.1, 52.8)	55.7 (50.3, 61.0)	0.31
Income					
<200% FPL	55.9 (53.8, 58.0)	62.1 (59.4, 64.7)	49.0 (44.8, 53.2)	38.6 (33.0, 44.4)	< 0.01
200–399% FPL	18.2 (16.6, 19.9)	16.3 (14.3, 18.4)	22.3 (19.0, 26.1)	20.6 (16.2, 25.9)	
≥ 400% FPL	25.9 (24.2, 27.7)	21.7 (19.5, 24.0)	28.6 (25.1, 32.4)	40.8 (35.6, 46.3)	
Education					
Less than HS	25.0 (23.2, 26.9)	31.8 (29.4, 34.4)	13.1 (10.0, 16.9)	5.5 (3.4, 8.6)	< 0.01
At least HS	25.6 (23.6, 27.6)	26.4 (23.9, 29.0)	29.5 (25.5, 33.7)	16.5 (12.1, 22.0)	
Some College	15.3 (13.9, 17.0)	14.5 (12.6, 16.6)	18.3 (15.0, 22.0)	15.1 (11.9, 19.0)	
College or more	34.1 (32.3, 36.0)	27.3 (25.0, 29.7)	39.2 (35.4, 43.1)	63.0 (57.3, 68.3)	
US Born	14.2 (12.9, 15.6)	11.8 (10.2, 13.6)	13.7 (11.4, 16.4)	23.9 (20.0, 28.4)	< 0.01
Body Mass Index					
< 23 kg/m^2^	43.2 (41.0, 45.4)	49.9 (47.0, 52.7)	27.5 (24.0, 31.3)	42.0 (36.1, 48.2)	< 0.01
23–27.4 kg/m^2^	40.0 (41.0, 42.2)	38.9 (36.2, 41.8)	41.8 (37.6, 46.1)	39.5 (34.0, 45.2)	
≥ 27.5 kg/m^2^	16.8 (15.2, 18.5)	11.2 (9.5, 13.1)	30.7 (26.7, 35.0)	18.5 (14.4, 23.3)	
Current drinker	37.2 (35.2, 39.2)	35.7 (33.1, 38.4)	32.1 (28.4, 36.0)	52.8 (47.1, 58.5)	< 0.01
Current smoker	11.6 (10.3, 13.0)	12.2 (10.5, 14.2)	9.9 (7.6, 12.7)	11.0 (8.4, 14.2)	0.34
Physical activity	68.1 (66.0, 70.1)	65.1 (62.3, 67.7)	72.2 (68.3, 75.8)	74.1 (68.3, 79.2)	< 0.01
NSES					
Low	37.1 (35.1, 39.2)	37.8 (35.1, 40.6)	30.7 (26.9,34.7)	42.7 (37.5, 48.2)	< 0.01
Medium	43.2 (41.1, 45.3)	46.1 (43.4, 48.9)	40.6 (36.4, 44.9)	35.1 (29.9, 40.7)	
High	19.7 (18.1, 21.4)	16.1 (14.3, 18.0)	28.8 (25.0, 32.9)	22.2 (17.3, 27.9)	

FPL: Federal poverty level; HS: High school; US: United States.

Chi-square p value to determine whether the distribution of each characteristic differs by Asian Sub-group.

**Fig. 1:**
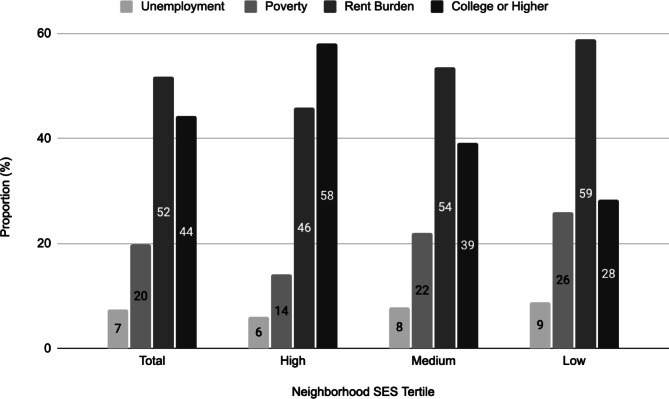
Neighborhood characteristics overall and according to neighborhood socioeconomic status factor score tertiles.

**Fig. 2:**
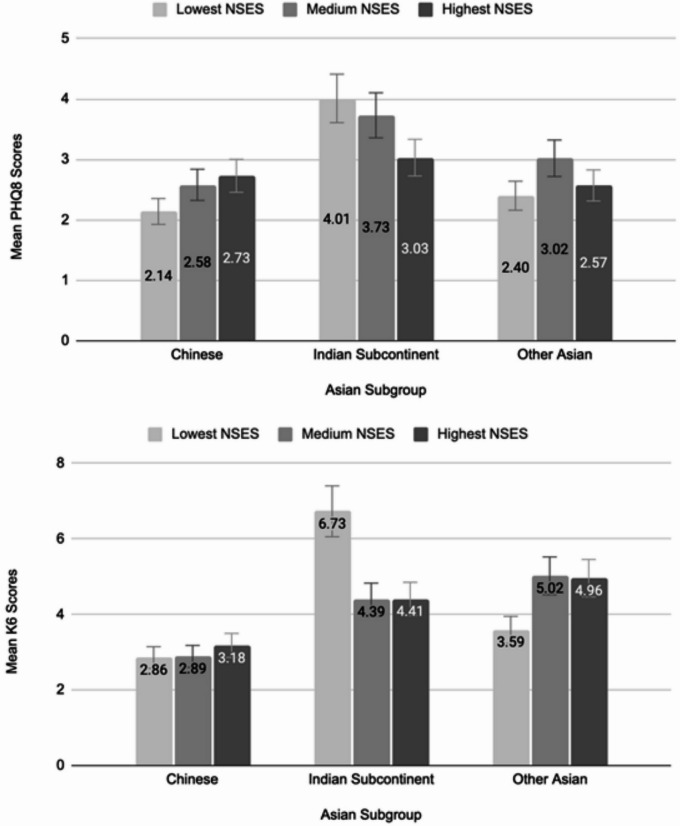
Mean PHQ8 and K6 by neighborhood socioeconomic status, among Asian American Adults, New York City Community Health Survey, 2018–2020.

The distributions of neighborhood level: unemployment, poverty, rent burden, and education, are shown in Fig. 1. Neighborhoods in the low NSES tertile had the highest rates of neighborhood level unemployment (8.8%), poverty (26.0%), high rent burden (58.9%) and the lowest rate of neighborhood college or greater education (28.4%). Neighborhoods in the high NSES tertile had the lowest rates of neighborhood level unemployment (6.1%), poverty (14.1%), high rent burden (45.8%), and the highest rate of college or greater education (58.1%). The mean PHQ8 score was 2.9 overall and was significantly lower in the Chinese group (2.6) compared with people from the ISC (3.6), *p* < 0.05. PHQ8 score did not differ by NSES overall or within any Asian sub-group (Fig. 2). The mean K6 score was 3.7 overall and was significantly lower in Chinese people (3.0) compared with ISC people (5.1) and with Other Asian people (4.8), p values < 0.05. K6 score did not differ by NSES overall. However, compared with high NSES, low NSES was associated with a higher K6 score among Asians from the ISC (6.73 vs. 4.41), and a lower K6 score among Other Asians (3.59 vs. 4.96), p values < 0.05.

Results from adjusted hierarchical linear regression models are shown in Table 2. There were significant interactions (*p* < 0.05) between NSES and Asian sub-group for both PHQ and K6. As a result, models were stratified by Asian sub-group. *For PHQ8*: in models adjusted for individual level age, sex, poverty, and education, low compared to high NSES was associated with a 0.65 lower PHQ8 score (95% CI: -1.10, -0.19) among Chinese people. After additional adjustment for nativity, BMI, current drinking, current smoking, and physical activity (model 2), results were attenuated but remained significant (beta: -0.57, 95% CI: -1.00, -0.13). *For K6*: in models adjusted for individual level age, sex, poverty, and education, low compared to high NSES was associated with a 1.27 higher K6 score among ISC people (95% CI: 0.59, 1.95). After additional adjustment for nativity, BMI, current drinking, current smoking, and physical activity (model 2), results were attenuated but remained significant (beta: 0.96, 95% CI: 0.10, 1.83). Associations were not significant among Other Asian people.

**Table 2:** Associations between neighborhood SES and mental well-being among Asian American residents of new York City, new York City community health Survey, 2018–2020.

	PHQ8 SCORE		K6 SCORE	
	Model 1	Model 2	Model 1	Model 2
	Beta (95% CI)	Beta (95% CI)	Beta (95% CI)	Beta (95% CI)
All Asians				
Low vs. high NSES	0.09 (− 0.41, 0.58)	0.14 (− 0.41, 0.68)	0.27 (− 0.46, 1.00)	0.33 (− 0.42, 1.07)
Medium vs. high NSES	0.19 (− 0.28, 0.67)	0.26 (− 0.24, 0.76)	− 0.01 (− 0.45, 0.43)	0.02 (− 0.41, 0.45)
Chinese				
Low vs. high NSES	− 0.65* (− 1.10, − 0.19)	− 0.57* (− 1.00, − 0.13)	− 0.13 (− 0.70, 0.44)	0.03 (− 0.61, 0.68)
Medium vs. high NSES	− 0.08 (− 0.60, 0.45)	− 0.11 (− 0.45, 0.66)	− 0.00 (− 0.43, 0.43)	0.08 (− 0.34, 0.50)
Indian Subcontinent				
Low vs. high NSES	0.34 (− 0.77, 1.44)	0.26 (− 0.87, 1.40)	1.27* (0.59, 1.95)	0.96* (0.10, 1.83)
Medium vs. high NSES	0.44 (− 0.67, 1.54)	0.28 (− 0.87, 1.40)	0.14 (− 0.51, 0.14)	− 0.12 (− 0.92, 0.67)
Other Asian				
Low vs. high NSES	0.07 (− 1.13, 1.27)	− 0.15 (− 1.15, 0.84)	− 0.67 (− 1.90, 0.57)	− 0.78 (− 1.97, 0.42)
Medium vs. high NSES	0.37 (− 0.56, 1.29)	0.54 (− 0.35, 1.44)	− 0.59 (− 1.75, 0.56)	− 0.60 (− 1.75, 0.56)

Model 1 was adjusted for: age, sex, individual level poverty and education.

Model 2 includes model 1 covariates in addition to: nativity, BMI, current drinking, current smoking and physical activity.

The models for “All Asians” do not include interaction terms. The interactions between Asian sub-group and NSES were significant for PHQ8 score and K6 (p values both < 0.05), models were stratified Asian sub-group.

Denotes statistical significance (p<0.05).

## Discussion

In a large population-based study representative of Asian American residents of NYC, we found that NSES was associated with psychological distress and depressive symptoms within certain Asian sub-groups. Among ISC people, residing in a neighborhood of low compared with high NSES was associated with greater levels of psychological distress. In contrast, among Chinese Americans, residing in a neighborhood of low compared with high NSES was associated with less depressive symptomatology. These findings underscore that the neighborhood context may exert influence on mental well-being. Further, our results show substantial heterogeneity in the association between NSES and mental health by Asian sub-group, emphasizing the importance of disaggregating Asian groups in research studies.

Low NSES has been shown to be associated with adverse mental health outcomes in prior studies^22^. Results from the longitudinal Waves of Midlife Study showed that living in a high compared with a low income neighborhood was associated with a lower odds of developing anxiety and depression^23^. In a recent study, refugee resettlement into a highly disadvantageous neighborhood was associated with an increase in risk of psychiatric disorders over 30 years^24^. Likewise, in a meta-analysis of assessing associations between the neighborhood social, natural, and built environment with mental health, a higher NSES was associated with a lower likelihood of pooled mental disorders^22^. Consistent with this previous research^22^, we found that low NSES is associated with psychological distress among Asian American adults from the ISC. There are numerous potential mechanisms which may explain this relationship. First, neighborhood of low SES may be characterized by poor conditions such as community disinvestment, decay, and poor safety, all of which can contribute to heightened psychological distress^25^. Stressors such as crime, noise, and disorder can contribute to mental disorders^22^. Poor neighborhood environments also have the potential to erode social resources and promote disconnection among its residents^26^. Second, in low SES neighborhoods, there are fewer green spaces and grocery stores relative to higher income communities^11^. Green spaces, such as parks or trails, have been associated with mental well-being^27,28^, particularly in urban areas^29^. In fact, as the number of green spaces and access to them increase, residents often report an improvement in mental health;^30^ this may be due to increased physical activity and greater opportunity for positive social interaction as a result of utilization of a green space, which in turn can improve mental well-being. Third, low NSES can be indicative of lower levels of health insurance, or lower quality health insurance^31^, both of which may limit individuals from adequate mental health resources and further contribute to poor mental well-being overall.

In contrast to our results among Asians from the ISC, we found that among Chinese Americans, low compared with high NSES was associated with less depressive symptomatology. While counterintuitive, these findings may be related to differences in item endorsement among Chinese Americans, particularly immigrants of lower SES. In fact, the concept of mental health/depression may be different among Chinese immigrants compared to other ethnic groups. First, Chinese immigrants underutilize mental health services more than the general population or US born Chinese Americans^32^. Second, cultural differences may impact the way that Chinese immigrants interpret aspects of mental health compared with other immigrant groups. One study found that three items (sleep, appetite and psychomotor disturbances) showed differences in item functioning, suggesting that, to Chinese Immigrants, the symptoms had a different meaning or were poorly understood compared to other US ethnic groups^33^. Such studies suggest that it is possible that Chinese immigrants are not endorsing questions related to depressive symptoms in the same way compared with other ethnic groups. This is further evidenced by a prior study that found suspiciously low PHQ-9 depression scores for Chinese immigrants, especially for men compared with Hispanic and non-Hispanic White and Black individuals^34^. However, we caution over-interpretation as more comparative studies are needed to make definitive claims. Further, additional research is needed to determine whether common study instruments can minimize or eliminate differential measurement bias, especially among lower SES Chinese American individuals. Another potential explanation for these findings may be related to our inability to properly account for social capital and social cohesion in our models^35^. Ethnic enclaves may provide a protective effect for some immigrant groups and we did not account for this in our analysis^14^. In fact, the Chinese American sub-group was more likely to have lower income, live in a lower NSES neighborhood, and more likely to be Foreign born compared with Asians from the ISC or Other Asians. Taken together, these studies may suggest reasons why we find an inverse association between NSES with depressive symptoms among Chinese residents of NYC.

Our current study has many notable strengths. To our knowledge this is the first study to assess the association between NSES with mental health among Asian Americans. It is conducted in a large sample of over 4,500 Asian adults and is representative of Asian American residents of NYC and includes data on disaggregated Asian American groups. However, this study also has some limitations. First, data collected in this study were self-reported during a telephone survey and therefore subject to self-reporting bias. Likewise, due to the cross-sectional design of the study, we could not establish temporality, as individuals often self-select their neighborhoods. Further, we did not have an adequate measure of time lived in the neighborhood or time spent out of the neighborhood (e.g., duration of exposure) since the survey did not measure movement within or across communities. While disaggregated data were available for Chinese Americans, due to limited sample size of Other Asian sub-groups, disaggregation of “Other” Asian people was not possible. Furthermore, the ISC sub-group included all participants who descended from the ISC, encompassing a broad range of different nations, and requiring disaggregation. There is also potential for uncontrolled confounding due to a limited set of factors measured. Notably, the survey is conducted in many languages including Cantonese and Mandarin, so not all Chinese persons with limited English proficiency were excluded from the sample. Only 14% of the study population was born in the US. While we adjusted for nativity status, we recognize that other information, unavailable, such as years residing in the US, is relevant and was not accounted for. Another limitation is the timeframe of the study, which includes the beginning of the COVID-19 pandemic. Asian Americans experienced unprecedented anti-Asian discrimination, especially in NYC^36^. This may have impacted mental well-being^37^, skewing results to show worse mental health outcomes than usual. We note that this limitation is only applicable for the K6 scale which was assessed in 2019–2020. Further, depressive symptoms were assessed prior to the COVID-19 pandemic in 2017–2018 and results for depressive symptoms were similar in direction to that of the psychological distress assessment. Additionally, though a factor loading threshold of 0.3 is generally accepted, caution is warranted as different academic fields of study utilize different conventions^15^. Finally, though census tract (or even zip code) level data is preferable, we were limited to geolinked data to the 55 community districts used to define the neighborhood due to privacy concerns.

## Conclusion

In a population-based study of NYC Asian American residents, we found that higher compared to lower NSES was associated with more depressive symptomatology among Chinese Americans but less psychological distress among Asians from the ISC Indian subcontinent. These results provide preliminary evidence of an association between NSES and mental health–– among certain Asian Americans sub-groups, identifying the neighborhood environment as a potential intervention point to improve mental well-being. Our research also highlights the heterogeneity of the Asian American population, which must be accounted for in health research. Future directions include working to understand why these trends exist and identifying interventions that can ameliorate the growing burden of mental illness in this fast-growing population.

## Data Availability

The data that support the findings of this study are available from the New York City Department of Health and Mental Hygiene, but restrictions apply to the availability of these data, which were used under license for the current study, and so are not publicly available. Data are however available from the corresponding authors upon reasonable request and with permission of the New York City Department of Health and Mental Hygiene.
